# Acupuncture for radiotherapy-induced nausea and vomiting: A systematic review protocol

**DOI:** 10.1097/MD.0000000000016027

**Published:** 2019-06-14

**Authors:** Yu Shi, Tao Xu, Qiutong Chen, Jing Wu, Yilin Zhong, Siping Song, Yang Chen, Wen Gao, Ling Zhao

**Affiliations:** College of Acupuncture and Moxibustion and Tuina, Chengdu University of Traditional Chinese Medicine, China.

**Keywords:** acupuncture, nausea, radiotherapy, systematic review, vomiting

## Abstract

**Background:**

Assessing the effectiveness and safety of acupuncture therapy for treating patients with radiotherapy-induced nausea and vomiting (RINV) is the main purpose of this systematic review protocol.

**Methods:**

The following electronic databases will be searched from inception to Sep 2019: Cochrane Central Register of Controlled Trials, PubMed, Web of Science, EMBASE, China National Knowledge Infrastructure, Traditional Chinese Medicine, Chinese Biomedical Literature Database, Wan-Fang Database, and Chinese Scientific Journal Database. All published randomized controlled trials in English or Chinese related to acupuncture for RINV will be included. The primary outcome is the severity and frequency of RINV during radiotherapy. The secondary outcomes is the physical condition and quality of life after radiotherapy. Two reviewers will conduct the study selection, data extraction, and assessment independently. The assessment of risk of bias and data synthesis will be conducted with Review Manager Software V.5.2.

**Results:**

The results will provide a high-quality synthesis of current evidence for researchers in this subject area.

**Conclusion:**

The conclusion of our study will provide evidence to judge whether acupuncture is an effective intervention for patients suffered from RINV.

**PROSPERO registration number:**

CRD42019130952.

## 1 Introduction

As one of the most common diseases in the world, cancer has significantly influence people's life and put a huge burden to the society. Radiotherapy (RT) is one of the main treatment modalities being used, in over 50% of all cancer patients, either alone or more commonly in combination with surgery and chemotherapy to treat a wide range of cancers.^[1]^ Considering the huge number of cancerous people, RT will impact these patients’ life a lot with its effects and side effects. Radiotherapy-induced nausea and vomiting (RINV), as one of the most common side effects during the RT, from which about 50% to 80% of the patients undergoing RT will suffer.^[2]^ RINV influenced terribly the quality of the patients’ life, like influencing patients’ ability to enjoy meals or imposing difficulty on the patients’ loved ones.^[3]^ What is worse, uncontrolled nausea and vomiting may result in patients delaying or refusing further RT, so it is very necessary to find a way of increasing the effect and decreasing the side effects of RT in maximum.^[4]^

For now, most of the patients will be suggested to take the antiemetic by the international antiemetic guidelines.^[5]^ This treatment is effective but produces some side effects as well like headache, sedation, dry mouth, constipation, and so on. A total of 77.1% of the patients with granisetron and 76.2% with ondansetron will suffer these adverse experience, which makes it an unsatisfying method to treat RINV.^[2,6,7]^

As a widely-used conventional therapy, acupuncture has been applied in various diseases in China and gets more and more popular all over the world.^[8]^ Although the mechanisms of acupuncture are not completely clear yet, a plenty of high-quality clinical trials have been conducted to prove that this therapy is effective and safe.^[9]^ For some symptoms, which caused by certain therapies, like nausea and vomiting, study shows that acupuncture could reduce nausea and vomiting induce by chemotherapy and RT.^[10,11]^ What is more, compared with antiemetic, acupuncture has less side effects, which could be the most important factor for patients to choose this method instead of the medication.

As there are plenty of processes and mechanisms involved in course the acupuncture taking effect, how does this therapy exactly work still remain unknown. And there are certain studies showing that the placebo effect may be the main mechanism resulting in relief of RINV with acupuncture.^[12]^ Neural mechanism like stimulating the secretion of endogenous opioid endorphin has been proved one of mechanisms of acupuncture therapeutical effect, but for RINV relative neural mechanisms have not been found yet.^[13]^ Besides of the results based on modern science, “deqi” sensation and regulating the yin and yang of human body are considered as the main mechanisms producing effects from acupuncture in the base of traditional Chinese medicine theory.^[14]^

Many systematic reviews have been conducted for the acupuncture treating chemotherapy-induced nausea and vomiting, but for RINV there have been few relative researches because nausea and vomiting caused by chemotherapy is more severe than by RT. As RINV has been a common problem for patients under RT, it is necessary to make a systematic review to provide a convincing conclusion whether acupuncture is an appropriate method to treat RINV.

## 2 Methods and analysis

### 2.1 Study registration

This systematic review protocol was registered with PROSPERO 2019 (registration number: CRD42019130952). And the protocol report is in the base of the preferred reporting items for systematic reviews and meta-analyses protocols (PRISMA-P) declaration guidelines.^[15]^ The review will be performed in line with the PRISMA declaration guidelines.^[16]^

### 2.2 Inclusion criteria for study selection

#### 2.2.1 Type of study

RCTs of acupuncture therapy for RINV without restrictions on publication status will be eligible for inclusion.

#### 2.2.2 Type of participant

Participants who were 18 years or older with RINV will be included in spite of gender, race, education, or economic status.

#### 2.2.3 Type of intervention

Acupuncture therapy, which includes manual acupuncture, body acupuncture, electroacupuncture, plum blossom needle, warm needling, and fire needling. Other methods, which includes transcutaneous electrical nerve stimulation, laser acupuncture, dry needling, cupping, and moxibustion will be excluded.

Comparison interventions, including sham acupuncture (including sham acupuncture at selected acupoints, sham acupuncture at non-acupoints, pseudo-acupuncture interventions, needling at inappropriate acupoints and nonpenetrating sham acupuncture), placebo, usual care, medication, no treatment, and other conventional therapies, will be included.^[17]^ In addition, the review of trials evaluating acupuncture combined with another treatment compared with other typical treatments alone will be included.

#### 2.2.4 Type of outcome measure

The primary outcome will be the severity and frequency after RT.^[18]^ The secondary outcomes will be the physical condition and quality of life after RT.

### 2.3 Search methods for identification of studies

#### 2.3.1 Electronic data sources

The following electronic databases will be searched from inception to September 2019: Cochrane Central Register of Controlled Trials, PubMed, Web of Science, EMBASE, China National Knowledge Infrastructure, Traditional Chinese Medicine, Chinese Biomedical Literature Database, Wan-Fang Database, and Chinese Scientific Journal Database. All published randomized controlled trials in English or Chinese related to acupuncture for RINV will be included.

#### 2.3.2 Searching other resources

The reference lists of potentially missing eligible studies will be scanned ant the relevant conference proceedings will be scanned as well.

### 2.4 Search strategy

The search strategy for PubMed is shown in Table 1. The following search keywords will be used: radiotherapy (eg, “radiotherapy” or “radiation therapy” or “radiation Treatment” or “targeted radiotherapy”); nausea (eg, “nausea”); vomiting (eg, “vomiting”); acupuncture (eg, “acupuncture” or “acupuncture therapy” or “body acupuncture” or “manual acupuncture” or “electroacupuncture” or “fire needling” or “plum blossom needling”; randomized controlled trial (eg, “randomized controlled trial” or “controlled clinical trial” or “random allocation” or “randomized” or “randomly” or “double-blind method” or “single-blind method” or “clinical trial.” The equivalent search keywords will be used in the Chinese databases.

**Table 1:**
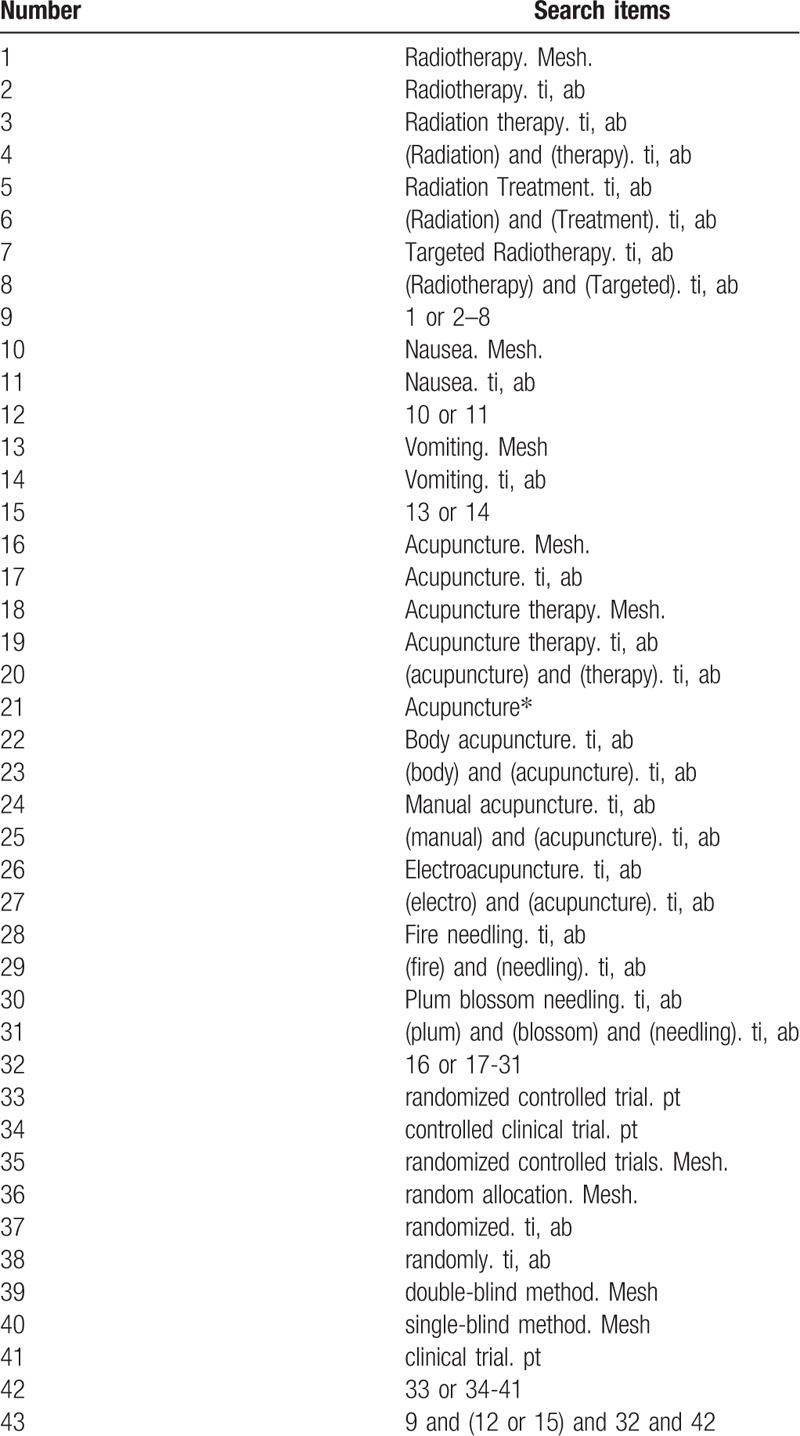
Search strategy for the PubMed database.

### 2.5 Data collection and analysis

#### 2.5.1 Selection of studies

**Figure 1:**
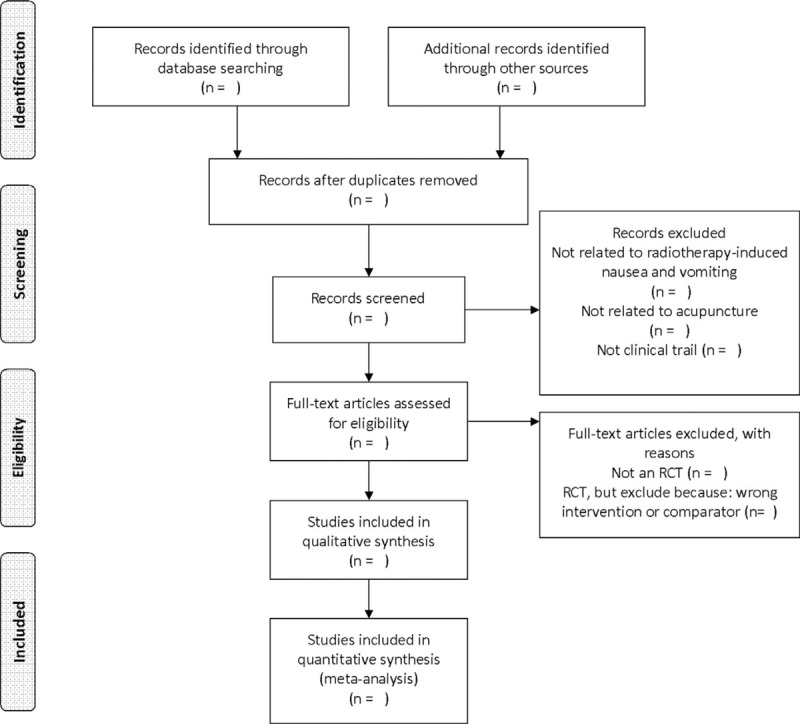
The PRISMA flow chart of the selection process. PRISMA = preferred reporting items for systematic review and meta-analysis.

The titles and abstracts of all searched studies will be reviewed and screened independently by 2 reviewers, aiming at identifying eligible trials and eliminating duplicated or irrelevant studies in line with the criteria; the full text of all possibly eligible studies will obtained if necessary. A discussion with the third reviewer is planned to solve the disagreements. A PRISMA flow diagram will be used to show the study selection process (Fig. 1).

#### 2.5.2 Data extraction and management

Before the data extraction, a standard data extraction form with following information will be created: year of publication, general information, country, participant characteristics, inclusion and exclusion criteria, sample size, methods, randomization, blinding methods, type of acupuncture interventions, control, outcome measures, results, adverse reactions, conflicts of interest, ethical approval, and other information. And these data will be extracted by 2 independent reviewers. A third reviewer will be set to be discussed with and judge the disagreements during the course. Contacting the authors for further information will be the solution for the insufficient data in the publications. Review Manager software (RevMan V.5.2.1) will conduct the analysis and synthesis after transferring all the data into the software.

#### 2.5.3 Assessment of risk of bias in included studies

Two reviewers will independently evaluate the risk of bias in all included studies with the Cochrane Collaboration's tool assessment method and complete the Standards for Reporting Interventions in Clinical Trials of Acupuncture checklist for the studies included.^[19]^ The following domains will be evaluated: selection bias, performance bias, attrition bias, detection bias, reporting bias, and other sources of bias. The assessments will then be divided into 3 levels: low risk, high risk, and unclear. Unclear or insufficient items will be obtained by contacting the corresponding author for further information. The third reviewer will be set to solve the disagreements.

#### 2.5.4 Measures of treatment effect

Dichotomous data will be presented as risk ratio and 95% confidence intervals (CIs), while continuous outcomes will be shown as standard mean difference 95% CI.

#### 2.5.5 Unit of analysis issues

The individual participant will the analytical unit.

#### 2.5.6 Management of missing data

Contacting the corresponding authors of the included studies will be the solution to obtain the missing or insufficient data of the primary results including sending emails or making a call. If missing data is not available, an intent-to-treat analysis will be performed as much as possible (the analysis should include data from all participants in the initially randomly assigned group) and a sensitivity analysis will be performed to determine if the results are inconsistent.

#### 2.5.7 Assessment of heterogeneity

*I*^2^ test will be used to quantify inconsistency and standard *χ*^2^ test will be used to detect statistical heterogeneity. Studies will be considered to have homogeneity if the *P* value exceeds .1 and the *I*^2^ value is less than 50%, and the fixed-effects model will be used. While studies will be considered to have significant statistic heterogeneity if the *P* value is less than .1 or the *I*^2^ value exceeds 50%, and subgroup analysis will be used to explore the possible cause. And the random-effects model will be applied if the heterogeneity is still important.

#### 2.5.8 Assessment of reporting biases

Funnel plots will be used to assess the reporting biases if more than 10 studies are included.

### 2.6 Data synthesis

RevMan V.52 will be used to perform the data synthesis, if it is possible carry out a meta-analysis. If no substantial statistical heterogeneity is detected, the data synthesis will be processed with the fixed-effects model, and if substantial statistical heterogeneity is detected, the data synthesis will be performed with the random-effects model. Possible reasons will be searched from a clinical and methodological perspective if different studies exist significant heterogeneity, and descriptive analysis or subgroup analysis will be provided. If there is no substantial heterogeneity between 2 studies, descriptive analysis will be conducted.

### 2.7 Subgroup analysis

Subgroup analysis will be conducted if the data are sufficient, according to the factors different outcomes and different control interventions.

### 2.8 Sensitivity analysis

Sensitivity analyses will be performed to evaluate the impact of sample size, study design, methodological quality and the effect of missing data, and to verify the robustness of the review conclusions if possible. The analysis will be repeated after low-quality studies are excluded.

### 2.9 Grading the quality of evidence

The grade of recommendations assessment, development and evaluation will be the tool to evaluate the quality of the evidence.^[20]^ Limitation of study design, inconsistency of results, indirectness, imprecision, and publication bias will be assessed. The assessments will be divided into 4 levels: very low, low, moderate, or high.

### 2.10 Ethics and dissemination

Formal ethical approval is not necessary as the data cannot be individualized. The results of this protocol will be disseminated in a peer-reviewed journal or presented at relevant conferences. The essential protocol amendments will be documented in the full review.

## 3 Discussion

This systematic review will assess the effectiveness of acupuncture for RINV. There are 4 sections in the review: identification, study inclusion, data extraction, and data synthesis. This review will help the doctors to choose acupuncture as an alternative treatment for RINV patients, and offer the patients more options to relieve their symptoms.

## Author contributions

YS, TX, and QTC mainly contributed to this manuscript and joint first authors. LZ obtained funding. JW, YLZ, and SPS drafted the protocol. YC and WG make the search strategy and it will be conducted by them. JW and YLZ will obtain copies of the studies and YC and WG will screen the studies to be included. Data extraction from the studies will be done by SPS and YLZ. JW and YLZ will put the data into RevMan. Analyses will be conducted by WG and YC and JW will interpret them. YS, TX, and QTC will draft the final review and WG and LZ will update the review. LZ will act as an arbiter in the study selection stage. All authors have read and approved the final manuscript.

**Data curation:** Yilin Zhong, Siping Song, Wen Gao.

**Funding acquisition:** Ling Zhao.

**Investigation:** Yang Chen.

**Methodology:** Yang Chen.

**Software:** Jing Wu, Yilin Zhong, Wen Gao.

**Supervision:** Ling Zhao.

**Visualization:** Siping Song.

**Writing – original draft:** Yu Shi, Tao Xu, Qiutong Chen.

**Writing – review and editing:** Yu Shi, Tao Xu, Qiutong Chen, Ling Zhao.

## References

[1] Delaney G Jacob S Featherstone C The role of radiotherapy in cancer treatment: estimating optimal utilization from a review of evidence-based clinical guidelines. Cancer 2005;104:1129–37. 16080176 10.1002/cncr.21324

[2] Feyer PC Maranzano E Molassiotis A Radiotherapy-induced nausea and vomiting (RINV): MASCC/ESMO guideline for antiemetics in radiotherapy: update 2009. Support Care Cancer 2011;19 Suppl 1:S5–14. 20697746 10.1007/s00520-010-0950-6

[3] Yee C Drost L Zhang L Impact of radiation-induced nausea and vomiting on quality of life. Support Care Cancer 2018;26:3959–66. 29808378 10.1007/s00520-018-4286-y

[4] Feyer PCh Maranzano E Molassiotis A Radiotherapy-induced nausea and vomiting (RINV): antiemetic guidelines. Support Care Cancer 2005;13:122–8. 15592688 10.1007/s00520-004-0705-3

[5] Feyer P Jahn F Jordan K Prophylactic management of radiation-induced nausea and vomiting. BioMed Res Int 2015;2015:1–8. 10.1155/2015/893013 PMC4573874 26425557

[6] Ruhlmann CH Jahn F Jordan K 2016 updated MASCC/ESMO consensus recommendations: prevention of radiotherapy-induced nausea and vomiting. Support Care Cancer 2017;25:309–16. 27624464 10.1007/s00520-016-3407-8

[7] Smith HS Cox LR Smith EJ 5-HT3 receptor antagonists for the treatment of nausea/vomiting. Ann Palliat Med 2012;1:115–20. 25841471 10.3978/j.issn.2224-5820.2012.07.07

[8] Zhou J Peng W Li W Acupuncture for patients with Alzheimer's disease: a systematic review protocol. BMJ Open 2014;4:e005896. 10.1136/bmjopen-2014-005896 PMC4139638 25142265

[9] Zhang Y Lin L Li H Effects of acupuncture on cancer-related fatigue: a meta-analysis. Support Care Cancer 2018;26:415–25. 29128952 10.1007/s00520-017-3955-6

[10] Enblom A Johnsson A Hammar M Acupuncture compared with placebo acupuncture in radiotherapy-induced nausea – a randomized controlled study. Ann Oncol 2012;23:1353–61. 21948812 10.1093/annonc/mdr402

[11] Ezzo JM Richardson MA Vickers A Acupuncture-point stimulation for chemotherapy-induced nausea or vomiting. Cochrane Database Syst Rev 2006;23:7188–98. 10.1002/14651858.CD002285.pub2 16625560

[12] Enblom A Lekander M Hammar M Getting the grip on nonspecific treatment effects: emesis in patients randomized to acupuncture or sham compared to patients receiving standard care. PLoS One 2011;6:e14766. 21448267 10.1371/journal.pone.0014766 PMC3063156

[13] Pomeranz B Acupuncture analgesia—basic research[M]//Clinical Acupuncture. 2001;Berlin, Heidelberg: Springer, 1–28.

[14] Liu S Zhou W Ruan X Activation of the hypothalamus characterizes the response to acupuncture stimulation in heroin addicts. Neurosci Lett 2007;421:203–8. 17574746 10.1016/j.neulet.2007.04.078

[15] Shamseer L Moher D Clarke M Preferred reporting items for systematic review and meta-analysis protocols (PRISMA-P) 2015: elaboration and explanation. BMJ 2015;349:g7647. 10.1136/bmj.g7647 25555855

[16] Liberati A Altman DG Tetzlaff J The PRISMA statement for reporting systematic reviews and meta-analyses of studies that evaluate health care interventions: explanation and elaboration. PLoS Med 2009;6:e1000100. 19621070 10.1371/journal.pmed.1000100 PMC2707010

[17] Jiang Y Yin L Wang Y Assessments of different kinds of sham acupuncture applied in randomized controlled trials. J Acupunct Tuina Sci 2011;9:199.

[18] Zhang J Su J Wang M The sensorimotor network dysfunction in migraineurs without aura: a resting-state fMRI study. J Neurol 2017;264:654–63. 28154971 10.1007/s00415-017-8404-4

[19] Higgins JP Altman DG Sterne J Assessing risk of bias in included studies. Cochrane handbook for systematic reviews of interventions: Cochrane book series. 2008:187–241.

[20] Guyatt GH Oxman AD Vist GE GRADE: an emerging consensus on rating quality of evidence and strength of recommendations. BMJ 2008;336:924–6. 18436948 10.1136/bmj.39489.470347.AD PMC2335261

